# A step further in our commitment to support early career researchers (ECRs)

**DOI:** 10.1002/2211-5463.70187

**Published:** 2026-01-05

**Authors:** Sara Fuentes, Miguel A. De la Rosa

**Affiliations:** ^1^ FEBS Open Bio Editorial Office Cambridge UK; ^2^ Institute for Chemical Research (IIQ), Scientific Research Centre Isla de la Cartuja (cicCartuja), Universidad de Sevilla‐CSIC Spain

## Abstract

As a not‐for‐profit open access journal, *FEBS Open Bio* is proud to support the scientific community not only through the publication of open access articles available to all but also through the charitable activities of FEBS. In this editorial, we look back at some of the highlights of the past year and look forward to the new initiatives planned for 2026—all aimed at supporting our community, the scientific community.

As one of the four not‐for‐profit journals owned by the Federation of European Biochemical Societies (FEBS), all net income from *FEBS Open Bio* is reinvested in the scientific community by supporting the charitable activities of FEBS. *FEBS Open Bio* is particularly proud of its longstanding support to early‐career researchers (ECRs), and in 2025, we were proud to award over 35 *FEBS Open Bio* Poster and Oral presentation prizes to ECRs at 30 international meetings and conferences, including two speed‐talk prizes and a poster prize at the 49th FEBS Congress, and an oral presentation prize and a poster prize at the 24th FEBS Young Scientists' Forum (YSF 2025). Details of all poster and oral presentation prize winners from 2021 onward can be viewed on the journal's website here: https://febs.onlinelibrary.wiley.com/hub/journal/22115463/febs-congress.

*FEBS Open Bio* is also proud to support the annual FEBS Congress by publishing the abstracts book as a supplement [1, 2], awarding poster and speed‐talk prizes at the event, and providing a fee waiver and a travel bursary to an ECR to attend the congress, as part of the *FEBS Open Bio* Article Prize. This prize is awarded to an ECR who has authored a paper of special interest published in the journal in the last 18 months. The 2025 *FEBS Open Bio* Article Prize was awarded to Giulia Lunghi (University of Milano, Segrate, Italy) as the first author of the outstanding paper ‘GM1 ganglioside exerts protective effects against glutamate‐excitotoxicity via its oligosaccharide in wild‐type and amyotrophic lateral sclerosis motor neurons’ [3]. The winning paper was selected by a jury comprised of three members of the journal's Editorial Board: Cláudio M. Soares (ITQB NOVA, Universidade Nova de Lisboa, Oeiras, Portugal), Ivana Novak (School of Medicine, University of Split, Croatia), and Jan Potempa (Microbiology Department, Jagiellonian University Krakow, Kraków, Poland). As part of the prize, Giulia received a travel and accommodation bursary to attend the 49th FEBS Congress in Istanbul and had the opportunity to present her work at the congress.

As part of our effort to highlight and promote the work of young researchers from the molecular life sciences disciplines, since 2023, *FEBS Open Bio* also publishes the abstracts and meeting report for the FEBS‐IUBMB‐ENABLE International Molecular Biosciences PhD and Postdoc Conference. The third FEBS‐IUBMB‐ENABLE conference, entitled ‘ARTIFICIAL INTELLIGENCE Reshaping biomedical and healthcare research’, was held at the Lee Kong Chian School of Medicine (LKCMedicine), Nanyang Technological University, Singapore from 4 to 6 December 2024 [4, 5]. We invite you to read the meeting report and check the abstracts. The fourth FEBS‐IUBMB‐ENABLE conference, entitled ‘BRIDGING MINDS – Interdisciplinary research for the future of life sciences’, was held at the CRUK Scotland Institute in Glasgo and the 2026 meeting will take place at the Jagiellonian University, in Krakow under the title ‘The code of life: Beyond the genome to multilayer biology’. Further information can be found at https://febs-iubmb-enableconference.org/.

## Content highlights: ‘In the Limelight’ Special Issues

Last year, *FEBS Open Bio* continued to publish comprehensive and critical reviews in topical research areas through the publication of ‘In the Limelight’ Special Issues. In 2025, we published five Special Issues on the themes of Education, Structural Bioinformatics, Research Protocols, Non‐coding RNAs in Cancer, and Tumor‐Stroma Interactions.

Luciane V Mello (Bioscience Education, University of Liverpool, UK; Member of the FEBS Education and Training committee and *FEBS Open Bio* Education section editor) and Ferhan Sağin (Department of Medical Biochemistry, Ege University Medical School, Izmir, Türkiye and Chair of the FEBS Education and Training committee) served as guest editors for our first special issue of the year which focused on education in biochemistry and the molecular sciences. This issue, which was conceived in close collaboration with the FEBS Education and Training Committee, was published as part of *FEBS Open Bio's* mission to disseminate advice on education techniques and resources. The issue included a White Paper from the inaugural FEBS Education and Training Conference (Antalaya, Turkey, 2024), selected abstracts from the conference, and three Review articles focused on student‐centered approach for assessment in Higher Education, teamwork promotion, and the impact of recent technological advancements in biomedical education. The White Paper published to mark the first FEBS Education and Training Conference summarizes key insights, discussions, and recommendations from the meeting and proposed actionable strategies to address some of the most pressing challenges in biochemistry and molecular sciences education [6]. In the first Review article, Rutherford *et al*. [7] discuss the observed shift in assessment and feedback practices in Higher Education, and suggest the use of the ‘Equity, Agency, Transparency’ (‘EAT’) framework as a basis for a more inclusive and meaningful assessment. The second Review article by Francis *et al*. [8] examines current approaches to assess teamwork and highlights several strategies for enhancing teamwork effectiveness. In the final Review article of this issue, Harris and Kazdağlı [9] summarize the key emerging trends and innovations in biomedical education, and how these might shape the future of biomedical education and training.

Our second ‘In the Limelight’ Special Issue of 2025 was guest edited by Cláudio M. Soares (ITQB NOVA, Universidade Nova de Lisboa, Oeiras, Portugal and *FEBS Open Bio* Editorial Steering Committee member) and Diana Lousa (ITQB NOVA, Universidade Nova de Lisboa, Oeiras, Portugal). This issue comprised five Review articles focused on recent advancements in the multidisciplinary field of structural bioinformatics. In the first article, Rosignoli *et al*. [10] examine how AlphaFold has shaped the current landscape of structural bioinformatics and explore future AI‐driven developments and opportunities. In the second Review article, Chaves *et al*. [11] discuss different computational protein–protein interaction design strategies with particular emphasis on antibody mimetic design. In the third article, El Salamouni *et al*. [12] examine the current driving forces behind synthetic nanobodies’ design and optimization. In the fourth article, Iglesias *et al*. [13] provide a comprehensive review of the different therapeutic and biotechnological applications of peptides, with special emphasis on the importance of structural information. In the final article of this Special Issue, Valério *et al*. [14] discuss the contribution of molecular simulation in the current understanding of viral entry mechanisms.

In April 2025, we published the third Special Issue of the year to highlight the role that Research Protocols play in ensuring research reproducibility, validity, and applicability. This ‘In the Limelight’ Special Issue was guest edited by Ivana Novak (School of Medicine, University of Split, Croatia and *FEBS Open Bio* Editorial Steering Committee member). It featured eight Research Protocols on X‐ray crystallography, enzymatic activity quantification, selective autophagy flux analysis, and frailty evaluation in mice. In the first Research Protocol, Pachl *et al*. [15] describe a pipeline for structure determination based on serial crystallography and a new microfluidic device. In the second article, Kaščáková *et al*. [16] provide a detailed procedure for co‐crystallization and soaking to achieve protein–ligand complex formation. In the third Research Protocol, Schönherr *et al*. [17] present the detailed protocol for their InCellCryst pipeline [18] to produce microcrystals within living insect cells. Bitala and colleagues [19] describe a fast, effective, and versatile protocol for baculovirus‐mediated multiprotein co‐expression in insect cells in the fourth article of this collection. The fifth Research Protocol, by Cezar *et al*. [20] provides a comprehensive procedure for quantifying surface‐displayed β‐lactamase on the cell wall of yeast. In the sixth article, Živković *et al*. [21] propose a protocol to quantify the enzymatic activity of the enzymes that activate substrates at the expense of ATP. In the seventh protocol, Marinković *et al*. [22] describe a simple flow cytometry quantification method for fluorescently tagged receptors of selective autophagy. In the final article of this Special Issue, Mladenovic and Pracer [23] provide a detailed description of a unique physical‐cognitive frailty score to measure frailty in rodents.

Our fourth Special Issue of 2025 was guest edited by Marcin Majka (Department of Transplantation, Institute of Pediatrics, Jagiellonian University, Krakow, Poland). This Special Issue highlighted the role of noncoding RNAs in cancer diagnosis and treatment. The first Review article by Burenina *et al*. [24] emphasizes the role that liver‐specific long noncoding RNAs (lncRNAs) can play as diagnostic biomarkers and potential therapeutic targets. In the second Review article, Wojcik *et al*. [25] critically examine the dual nature of R‐loops as both vital cellular regulators and potential threats to genome integrity, focusing on the role of R‐loops as diagnostic and prognostic biomarkers. In the third Review article, Tran *et al*. [26] discuss the role of lncRNAs as therapeutic targets in head and neck squamous cell carcinoma and their clinical significance. The last of the Review articles details the emerging role of circular RNAs in modulating cytokine signaling pathways that regulate cancer development as reviewed by Joshi *et al*. [27].

Isabel Fabregat (TGF‐β and Cancer Group, Bellvitge Biomedical Research Institute (IDIBELL) and CIBEREHD, L'Hospitalet de Llobregat, Barcelona) guest edited our final ‘In the Limelight’ issue of 2025 which featured four Review articles on Tumor‐Stroma Interactions. The first Review article by Skandalis *et al*. [28] tackles the complex amalgam of structures that forms the matrix as well as the role of the tumor microenvironment in tumor maintenance and progression. In the second article, Rafik *et al*. [29] present a comprehensive overview of the available 3D *in vitro* models for the study of tumor‐stroma interactions. The third Review article by de la Jara Ortiz *et al*. [30] describes recent findings regarding the role played by the different cancer‐associated fibroblasts (CAF) subtypes, as well as the development of 3D models to study Tumor‐Stroma Mechanics *in vitro*. In the fourth and final Review, Bernardo *et al*. [31] discuss recent major advances in our understanding of tumor stromagenesis and CAF heterogeneity in both primary tumors and metastasis, with a special focus on non‐small cell lung cancer.

To accompany our latest ‘In the Limelight’ issue on Tumor‐Stroma Interactions, we are pleased to announce that a free *FEBS Open Bio* webinar will be held on February 10th at 16:00 CET. Guest editor Isabel Fabregat together with several of the contributing authors will discuss trending topics in Tumor‐Stroma Interaction research. Registration opens at: https://zoom.us/webinar/register/WN_RAXzasTLQ5GoNWsBfCkm5A#/registration. We look forward to welcoming you all!

## Transforming our boards

The rapid evolution of AI has drastically accelerated the pace of change in scientific publishing. *FEBS Open Bio* remains committed to supporting the scientific community and to ensure we respond efficiently to the rapidly changing publishing landscape, in 2025, we launched two new initiatives: the Editorial Steering Committee and Publishing Liaison Officers.

The Editorial Steering Committee was created to ensure that *FEBS Open Bio* not only responds rapidly to the needs and expectations of the scientific community but, more importantly, remains at the forefront of innovation. We hope that this committee, composed of a small number of editorial board members, will enable us to better connect with the community we serve by providing regular feedback and input. The Editorial Steering Committee is formed by:

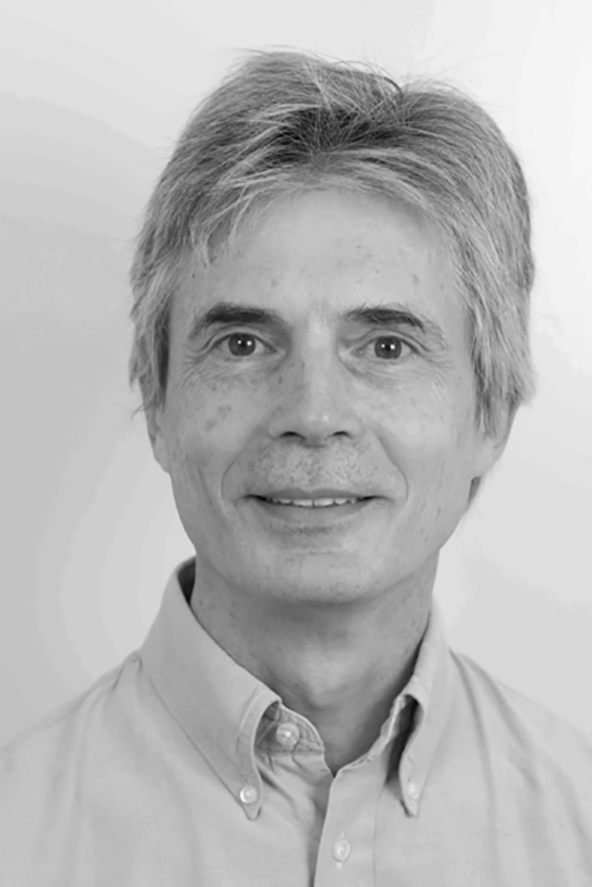

**Pierre Cosson**, Department of Cell Physiology and Metabolism, Université de Genève, Centre Medical Universitaire (CMU), Geneve, Switzerland

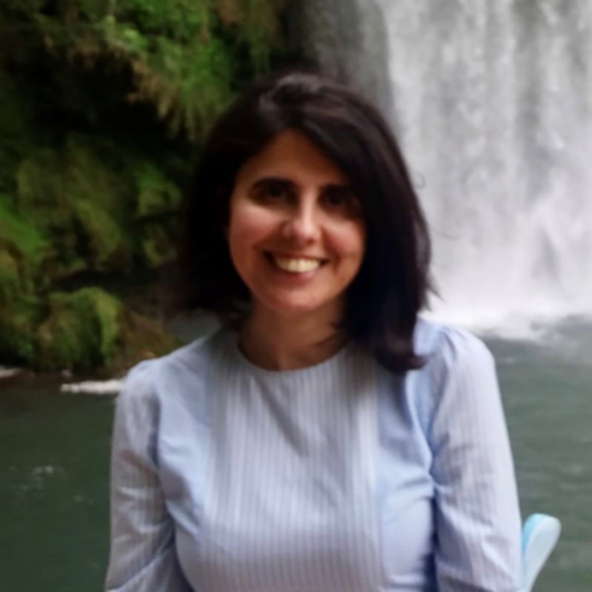

**Irene Díaz‐Moreno**, Institute of Chemical Research of the Scientific Research Centre Isla de la Cartuja—cicCartuja, Seville, Spain

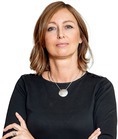

**Aleksandra Mladenovic**, Department of Neurobiology, Institute for Biological Research ‘Sinisa Stankovic’—National Institute of the Republic of Serbia, University of Belgrade, Belgrade, Serbia

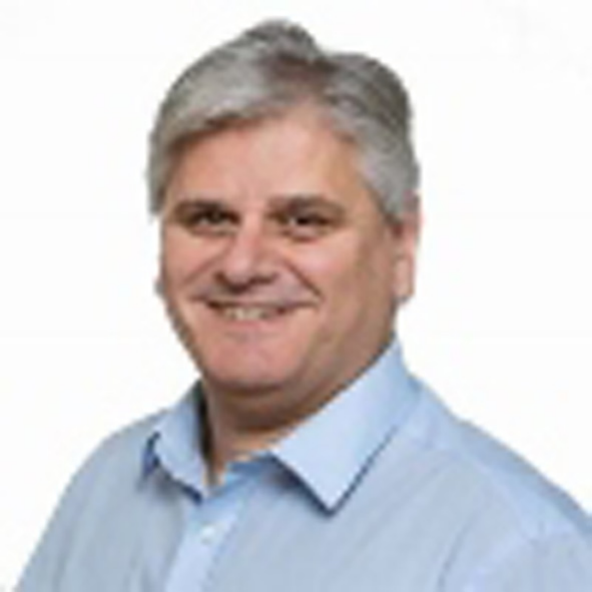

**Francesco (Frank) Michelangeli**, University of Chester, University of Birmingham, UK

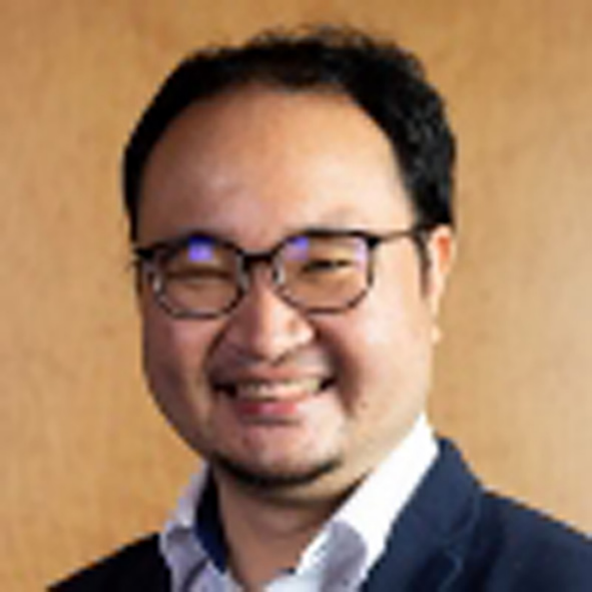

**So Nakagawa**, Department of Molecular Life Science, Tokai University School of Medicine, Kanagawa, Japan

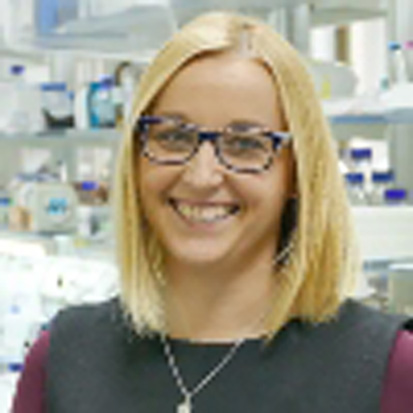

**Ivana Novak**, School of Medicine, University of Split, Croatia

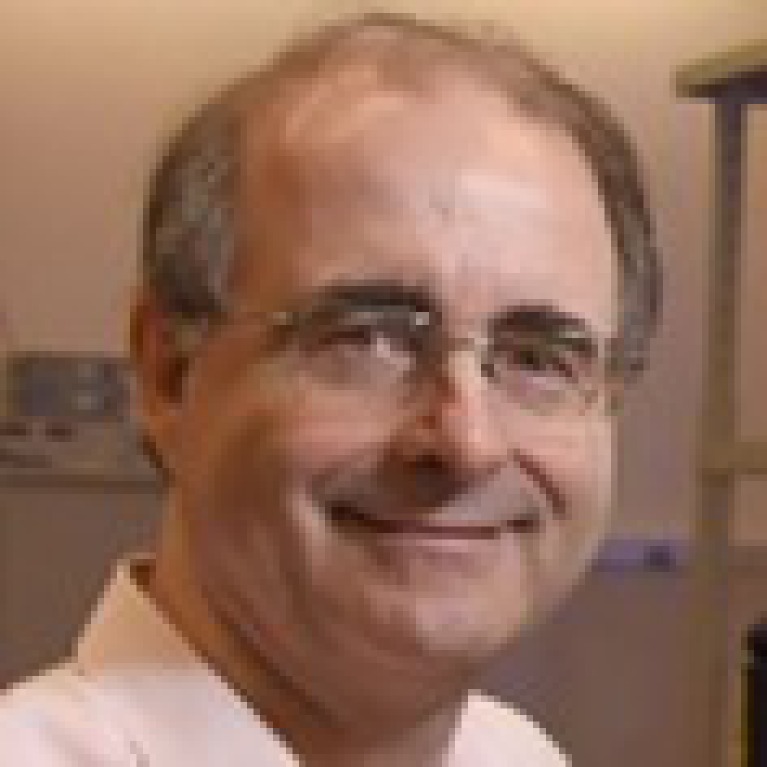

**Josep Rizo**, UT Southwestern Medical Center, Dallas, Texas, USA

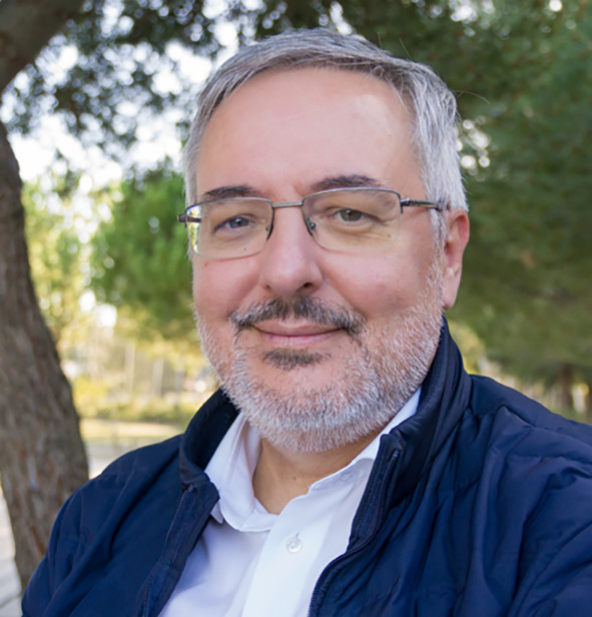

**Cláudio M. Soares**, ITQB NOVA, Universidade Nova de Lisboa, Oeiras, Portugal

Last year, we also launched the Publishing Liaison Officer role in order to bridge the gap between the journal and scientific researchers by serving as publishing advisors and working closely in collaboration with the journal team. In 2025, we were delighted to welcome six Publishing Liaison Officers to the journal from across the globe:

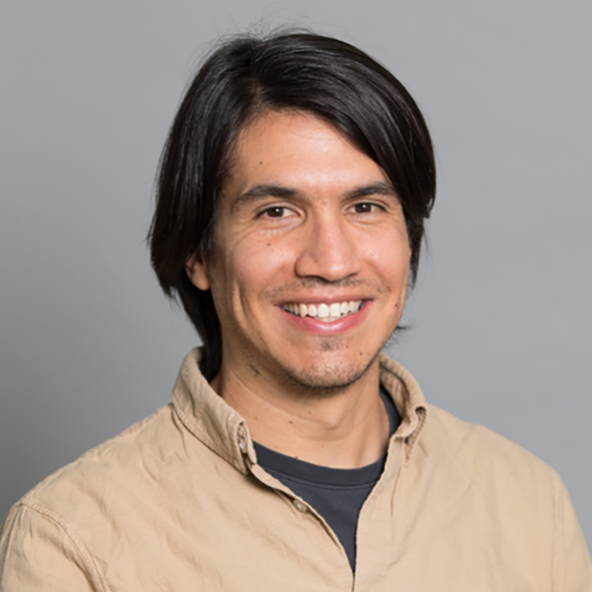

**Johnathan Canton**, Faculty of Veterinary Medicine, University of Calgary, Alberta, Canada

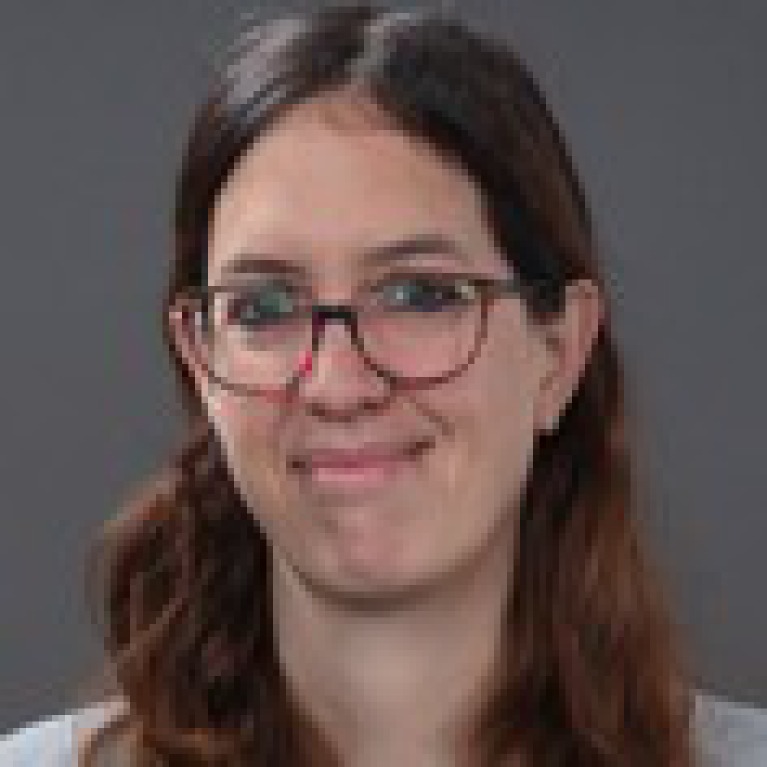

**Karla L. H. Feijs‐Žaja**, Institute of Biochemistry and Molecular Biology, RWTH Aachen University, Germany

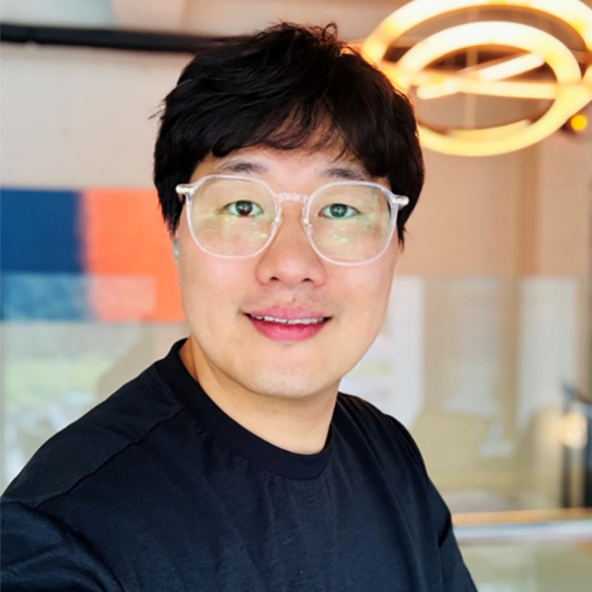

**Kyungtae Lim**, Department of Life Sciences, Korea University, Seoul, South Korea

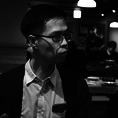

**Shoichi Sakaguchi**, Laboratory of Microbiology and Infection Control, Osaka Medical and Pharmaceutical University, Takatsuki, Osaka, Japan

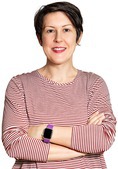

**Tamara Saksida**, Institute for Biological Research ‘Siniša Stanković’, National Institute of the Republic of Serbia, University of Belgrade, Belgrade, Serbia

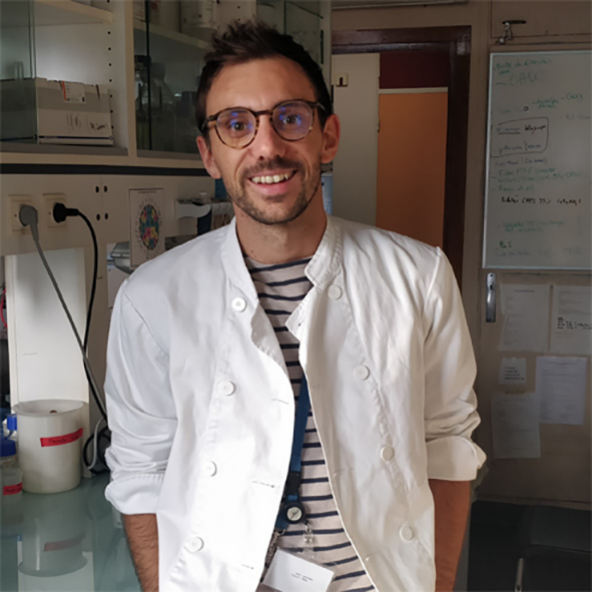

**Pierre Santucci**, CNRS Aix‐Marseille Université, Marseille, France

## Looking ahead to 2026: A step further in our commitment toward supporting ECRs

In 2022, we introduced Research Protocols with the aim to help combat the reproducibility crisis in life sciences. This year, we published the first ‘In the Limelight’ Special Issue focused on Research Protocols to highlight the importance of this article type. Reproducible, detailed and validated Research Protocols are vital at every career stage, but never more than when young scientists are getting acquainted with a new procedure. With this in mind, we are excited to announce our plan to publish two new ‘In the Limelight’ issues on Research Protocols on the topics of Photobiology and Fluorescence microscopy in 2026. We hope that these Special Issues, and more generally, *FEBS Open Bio* Research Protocols, will contribute to the success of many researchers’ experimental work.

We know that the support, advice, training and guidance received during early career years are paramount for the success of any researcher. Given the crucial role of peer review in scientific research, we believe it is important for ECRs to have the opportunity to actively participate in it. And, as with any skill, practice is necessary for improvement. To facilitate ECR participation in the peer‐review process, we are excited to announce the launch of the Early Career Reviewer hub, a program designed to involve ECRs in the peer‐review process of Research Protocols. We hope that through our focus on Research Protocols and the Early Career Reviewer hub launch, we continue to support and contribute to the professional development of early career molecular life scientists.

Finally, as we welcome 2026, we would like to express our gratitude to all the authors who have chosen *FEBS Open Bio* to publish their work, to all our editors, advisory board members, and reviewers who have made this possible and, last but not least, to all our readers for their support. We look forward to working with you in this coming year!

## Conflict of interest

The authors declare no conflict of interest.
